# Supplementary figures and images for: Analysis of modular gene co-expression networks reveals molecular pathways underlying Alzheimer’s disease and progressive supranuclear palsy
Source: PLoS One. 2022 Apr 14;17(4):e0266405. doi: 10.1371/journal.pone.0266405 (PMC9009680; doi:10.1371/journal.pone.0266405)

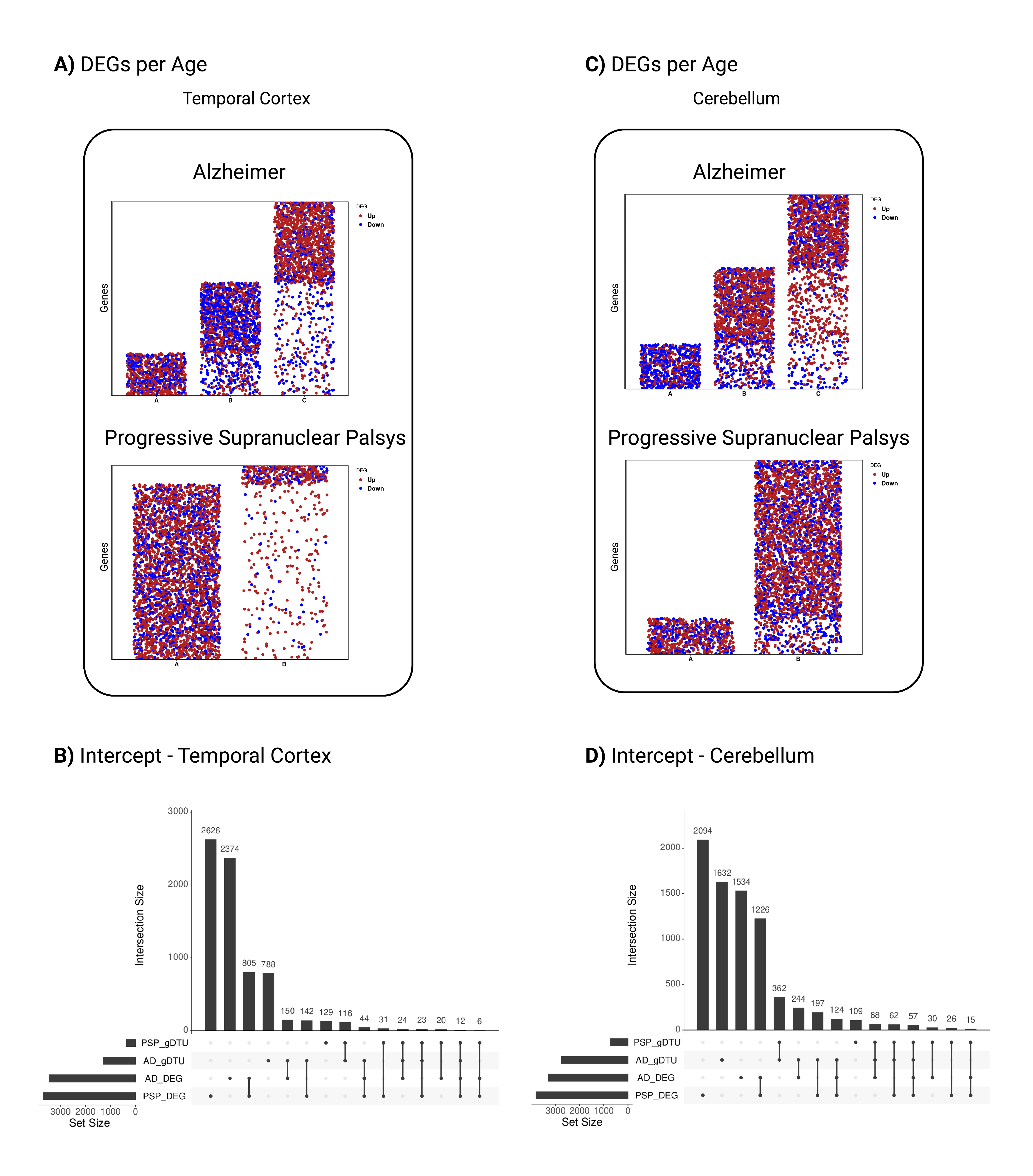

Supplement: S1 Fig — A) DEGs identified in temporal cortex data of AD and PSP. B) Intercept graphic showing the overlap among DEGs and gDTUS identified in the temporal cortex of AD and PSP patients compared to controls. C) Same as in (A) for cerebellum. D) Same as in (B) for cerebellum. Up (genes with higher expression when compared to control individuals), Down (genes with lower expression when compared to control individuals). (TIF) [file pone.0266405.s001.tif]

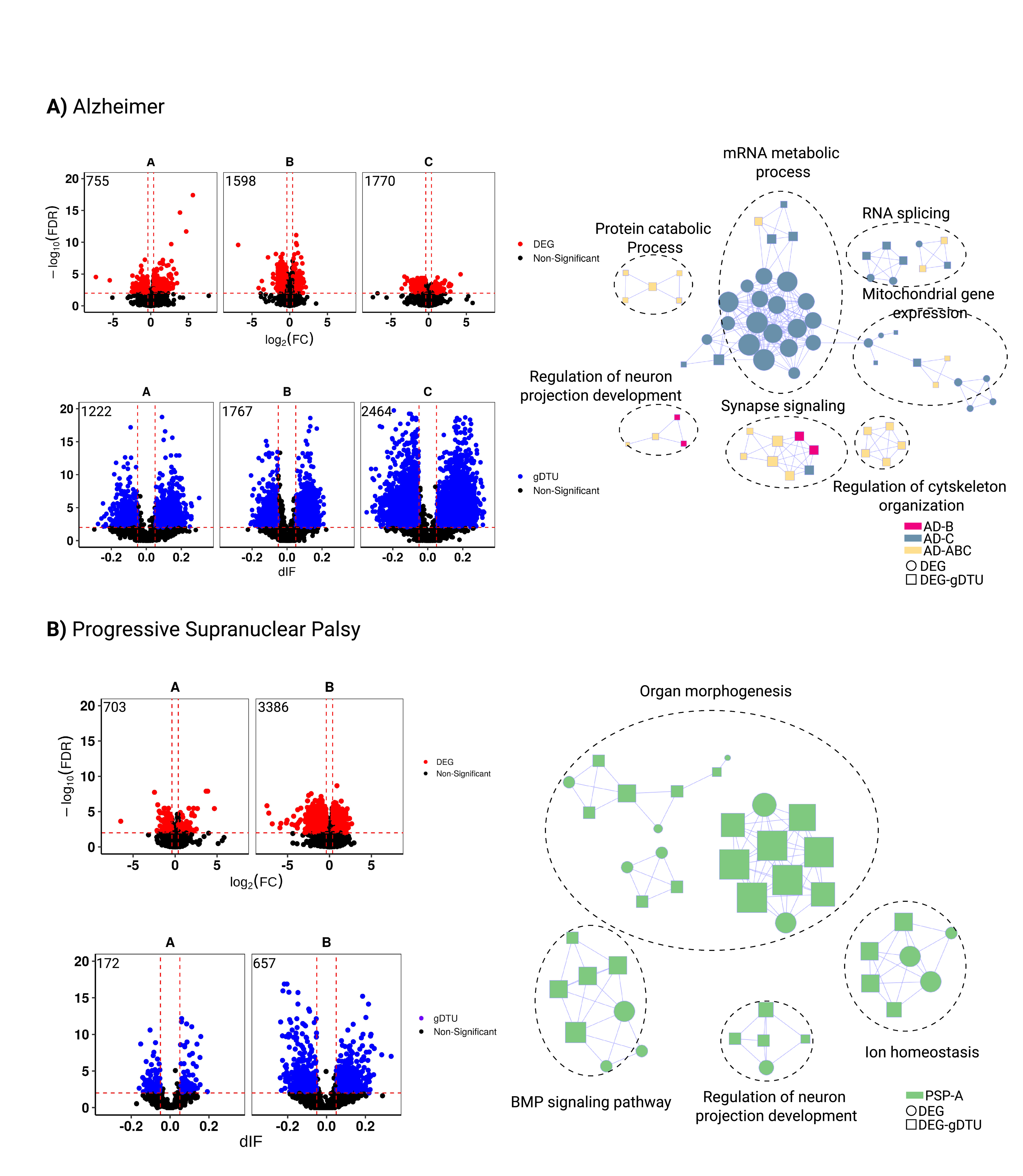

Supplement: S2 Fig — A) Volcano plots showing differentially expressed genes (DEGs, red dots; FC > 1.3 and FDR < 0.01), genes with differential transcript usage (gDTU, blue dots; Differential isoform fraction (dIF) and FDR < 0.01) and a network representation of gene ontologies (GOs) significantly enriched in AD. Circles, triangles and squares indicate, respectively, GOs enriched for DEGs, gDTUs or a combination of both. Colors in the network indicate groups where gene expression alterations were detected. B) Same for PSP. AD (Alzheimer), PSP (Progressive Supranuclear Palsy), A (age of death between 70–80 years old), B (age of death between 81–89 years old), C (age of death equal or superior to 90 years old), FDR (False Discovery Rate). Numbers of significantly altered genes identified in each analysis are shown in the top left corner of the volcano plots. (TIF) [file pone.0266405.s002.tif]

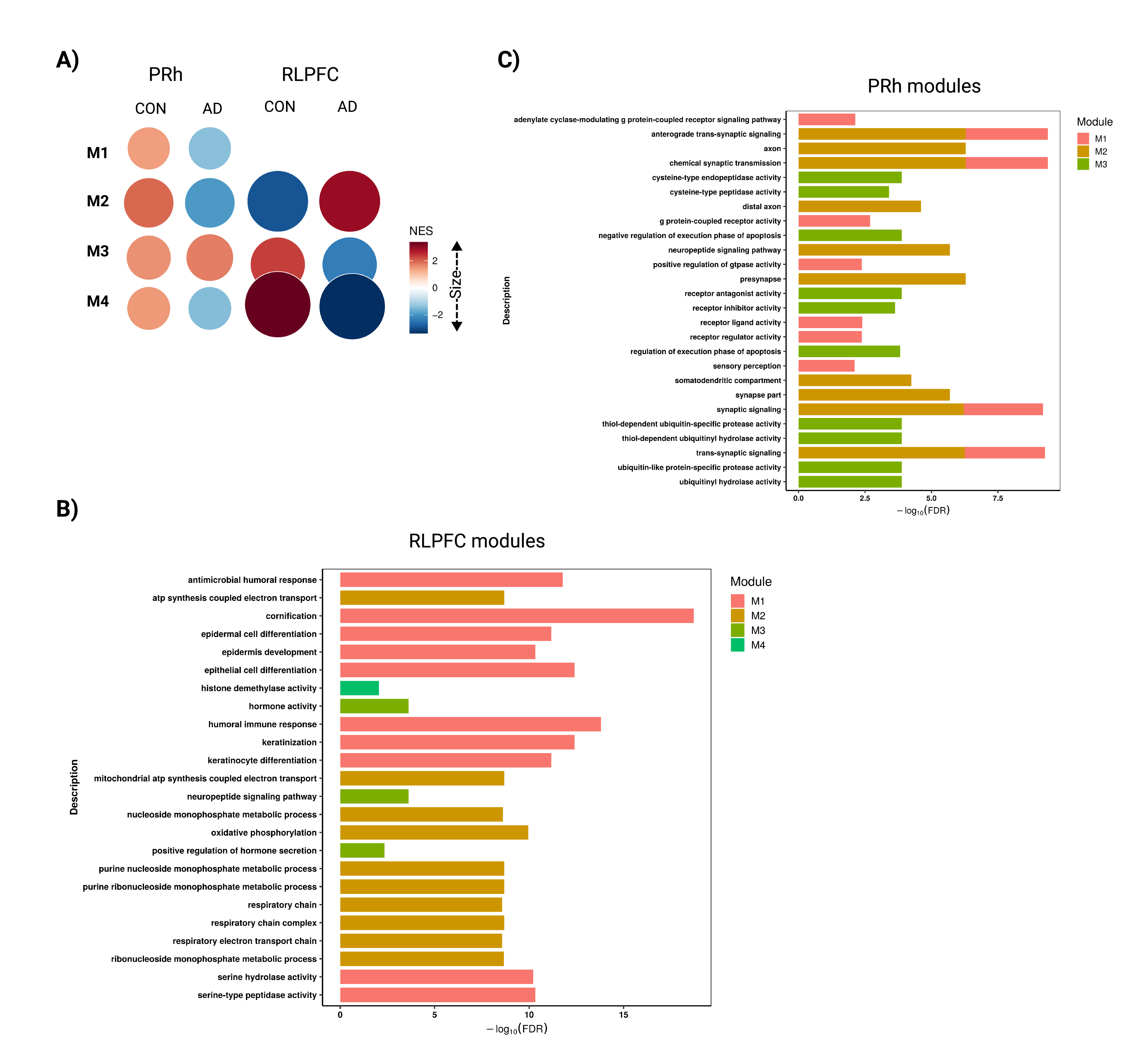

Supplement: S3 Fig — A) NES for modules identified in the PRh and RLPFC of AD patients compared to controls. Size of circles is equal to the absolute value of NES. Color of circles represents up (red) or down (blue) regulation between classes (AD and control) and is proportional to NES value. B-C) Over Representative Analysis (ORA) of RLPFC and PRh modules. Only ontologies with FDR < 0.01 are shown. (TIF) [file pone.0266405.s003.tif]

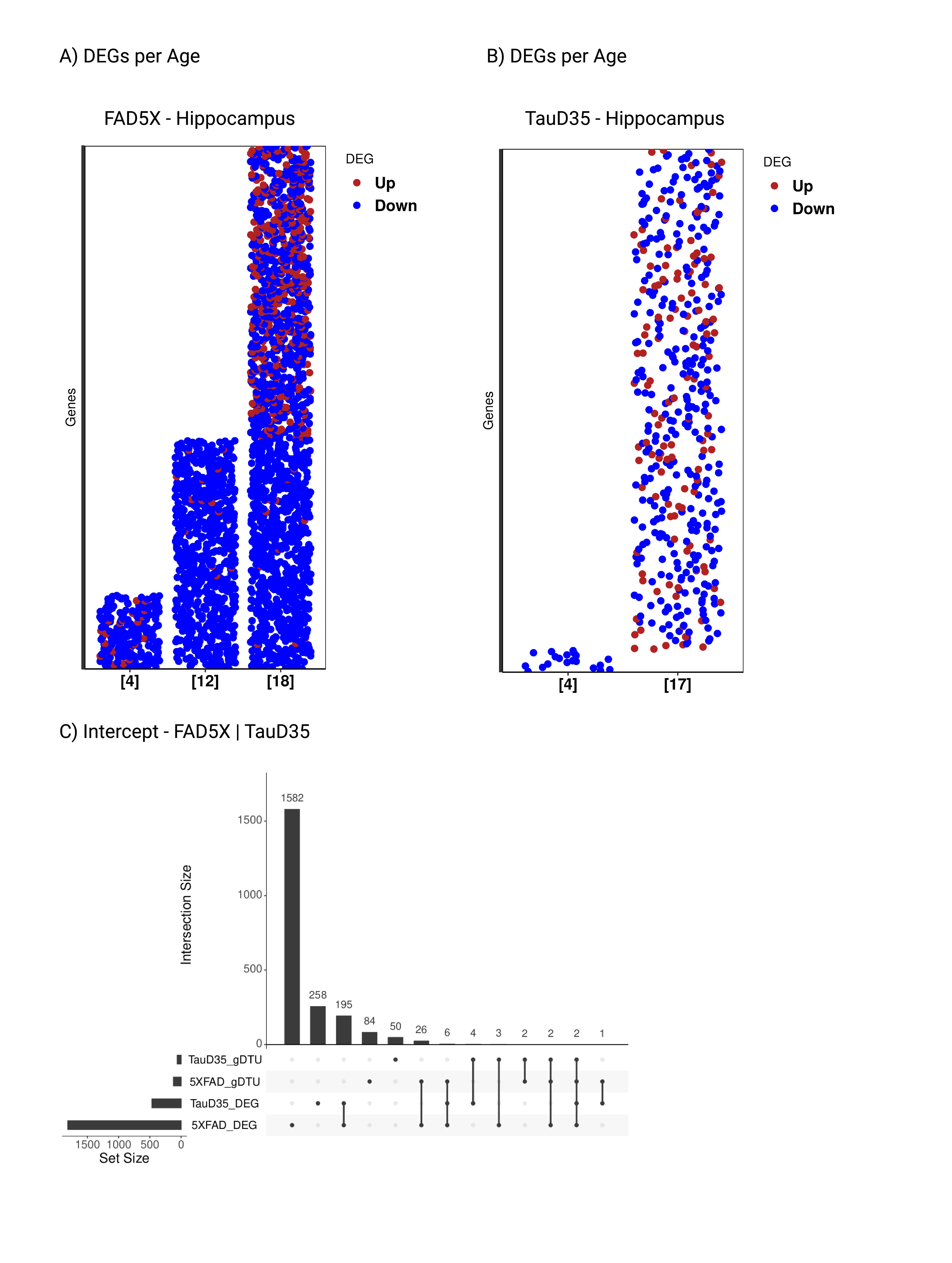

Supplement: S4 Fig — A-B) DEGs identified in the brains of 5XFAD (A) and TauD35 (B) compared to control animals. C) Intercept graphic showing the overlap among DEGs identified in the models. Up (genes with higher expression when compared to control animals), Down (genes with lower expression when compared to control animals). (TIF) [file pone.0266405.s004.tif]
